# Do incoming residents vary in measures of emotional status even prior to residency training?

**DOI:** 10.5116/ijme.62cb.f308

**Published:** 2022-07-29

**Authors:** Jeanne L. Jacoby, Amy B. Smith, Robert D. Barraco, Marna Rayl Greenberg, Elaine A. Donoghue, Bryan G. Kane, Jennifer E. Macfarlan, Lauren M. Crowley, Kevin R. Weaver, Joann Farrell Quinn

**Affiliations:** 1 Lehigh Valley Health Network, Department of Emergency and Hospital Medicine, University of South Florida Morsani College of Medicine (USF-MCOM), Schoenersville Road, Bethlehem, PA, USA; 2 Lehigh Valley Health Network, Department of Education, University of South Florida Morsani College of Medicine (USF-MCOM), Schoenersville Road, Bethlehem, PA, USA; 3 Lehigh Valley Health Network, Network Office of Research and Innovation, University of South Florida Morsani College of Medicine (USF-MCOM), Schoenersville Road, Bethlehem, PA, USA; 4 University of South Florida Morsani College of Medicine (USF-MCOM), Tampa Campus, Tampa, FL, USA

**Keywords:** Empathy, residents, specialty, emotional intelligence, burnout

## Abstract

**Objectives:**

To determine whether Empathy, Emotional Intelligence, and Burnout scores differ by specialty in incoming residents.

**Methods:**

This is a single-site, prospective, cross-sectional study. Three validated survey instruments, the Jefferson Scale of Physician Empathy, Maslach Burnout Inventory, and Emotional and Social Competency Inventory, were written into a survey platform as a single 125-question Qualtrics survey. Over three academic years, 2015-2017, 229 incoming residents across all specialties were emailed the survey link during orientation. Residents were grouped by incoming specialty with anonymity assured. A total of 229 responses were included, with 121 (52.8%) identifying as female. Statistical analysis was performed using the Analysis of Variance or Kruskal-Wallis test, Chi-Square or Fisher’s Exact test, and Independent Samples t-test or Mann Whitney U test. A Bonferroni correction was applied for pairwise comparisons.

**Results:**

Family Medicine had a higher median Jefferson Scale of Physician Empathy score (127) compared to Emergency Medicine (115), (U=767.7, p=0.0330). Maslach Burnout Inventory depersonalization and personal accomplishment subcategory scores showed a significant difference between specialties when omnibus tests were performed, but pairwise comparisons with emergency medicine residents showed no differences. Differences were found in the Maslach Burnout Inventory categories of Depersonalization (χ^2^_(8, N=229)_ =15.93, p=0.0434) and Personal Accomplishment level (χ^2^_(8, N=229)_ =20.80, p=0.0077) between specialties.

**Conclusions:**

Differences in measures of well-being exist across specialties, even prior to the start of residency training. The implication for educators of residency training is that some incoming residents, regardless of specialty, already exhibit troublesome features of burnout, and resources to effectively deal with these residents should be developed and utilized.

## Introduction

The importance of empathy, emotional intelligence (EI), and burnout among residents has received increasing attention in recent years. However, previous research efforts have been directed toward the development of these indicators of well-being during post-graduate (residency) medical training.^1^^-^^4^ The purpose of this study was to determine whether evidence of decreased empathy, measures of EI, and increased burnout exist even prior to the beginning of post-graduate training and whether they vary by medical specialty choice.

Studies assessing the mental health status of residents in multiple countries have shown that mental health issues and burnout indicators are much more prevalent than among the general populace.^1^^-^^3^ Key to reducing these conditions is identifying their underlying causes and providing the appropriate resources and coping techniques to residents suffering from mental health declines. Higher levels of empathy have been shown to strongly correlate with burnout resilience.^5^

It has been noted that in United States medical students, measures of empathy decline after the third year of medical school.^6^^-^^8^ The results of these investigations have been disparate, with important differences noted in medical students and residents as training progresses. Burnout has been noted in attending physicians.^9^^,^^10^ Lower levels of burnout and higher levels of empathy have been shown to positively affect patient perceptions of their physicians and even patient outcomes.^10^^,^^11^ Physicians with higher EI tend to have higher patient satisfaction scores. Weng and colleagues found that patients were more satisfied when their physician had less burnout and that EI and burnout were inversely correlated in 110 internists.^10^ A recent survey of 127 trauma patients six weeks after discharge from the hospital showed that subjective measures of medical treatment were higher when their physicians were more empathetic, suggesting that even on a surgical service (where outcomes could be considered objective) patients perceived they had better care when their physician had more empathy.^11^ With these disparate results, this study seeks to clarify and determine if there are significant patterns among specialties, with regard to burnout, EI, and empathy.

Previous investigations have failed to establish a consistent pattern regarding Burnout, Empathy, and EI as they relate to specific Graduate Medical Education (GME) training programs.^9^^,^^12^^,^^13^ Further, whether there is a relationship between specialty choice and EI is unclear.^14^^,^^15^ Here we report the cross-sectional intake results (for Post-Graduate Year-1s) of multiple GME training programs in a suburban healthcare network to determine if there is a significant difference and identify specialties that may need to devote increased resources to reducing burnout and increasing EI and empathy. Identifying and treating burnout as early as possible, especially among students and residents, helps to improve the overall quality of their education and knowledge base upon graduation.

## Methods

### Study design and participants

This single-site, prospective, cross-sectional study was conducted at a healthcare network located in Pennsylvania, USA that trains approximately 80 incoming residents per year in ten GME residency training programs. All incoming residents during three academic years (2015-2017) were offered the chance to enroll in the study. Participants were recruited by email and had to opt in to have their data utilized for research. No incentive was provided to complete the survey. The survey took about 20 minutes to complete. This study cohort included incoming residents from Surgery, Internal Medicine (IM), Emergency Medicine (EM), Pediatrics, Obstetrics and Gynecology (OB/GYN), Family Medicine (FM), Transitional (Tr), Osteopathic internship and Dentistry. Other GME programs, including fellowships for physicians, administrators, chaplains, and nursing, were not eligible for inclusion in the study. The study was approved by the Institutional Review Board (IRB).

### Data collection

To assess Empathy, EI, and Burnout, three previously validated instruments, the Jefferson Scale of Physician Empathy (JSPE), Maslach Burnout Inventory- Human Services Survey (MBI-HSS), and Emotional and Social Competency Inventory (ESCI), were administered as a single survey; licensing was obtained for all study instruments. Demographic data, including gender (male, female, other), age, marital status, degree(s) held, and program year, was collected. All three survey instruments use a Likert scale to rank the participant’s agreement level with a given statement. In total, the survey included 125 separate questions for the participants to respond to. The survey was administered via Qualtrics, a commercially available survey instrument. A link to the survey was emailed to the incoming interns, and a random number was assigned to each participant to preserve anonymity.

### Data analysis

Descriptive statistical analysis was conducted to summarize the resident population. Comparisons across specialties and years were conducted with the use of the Analysis of Variance (ANOVA) or Kruskal-Wallis test and the Chi-Square or Fisher’s Exact test. Post hoc pairwise comparisons were made between specialties when the omnibus test was statistically significant, and a Bonferroni correction was applied. Differences between genders were analyzed using the Chi-Square or Fisher’s Exact test for categorical data or an Independent Samples t-test or Mann-Whitney U test, depending upon the normality of the continuous variables. Analysis was conducted in SAS version 9.3.

## Results

There were 229 incoming residents in our network from June 2015 through 2017. All 229 residents were included in demographic, JSPE, and MBI-HSS analyses. Three were excluded from ESCI analyses due to an insufficient number of responses to their surveys. Between the three academic years, there were several statistically significant differences in respondent demographics and score patterns (Table 1).

Key differences were found among specialties in incoming residents’ emotional status. Results of the Kruskal-Wallis H test demonstrated a significant difference between JSPE scores and medical specialty (χ^2^_(8, N=229)_ =23.35, p=0.0029). Post hoc results using a Bonferroni correction identified a significant difference between FM and EM residents in JSPE scores, as FM had a higher score (Mdn=127) compared to EM (Mdn=115) (U=767.7, p=0.0330).

**Table 1:** Demographics and survey scores for all interns and distributed by academic year

Characteristic	Total (n=229)	2015 (n=83)	2016 (n=72)	2017 (n=74)	p-value
Age, years median (IQR)	27.0 (26.0-28.0)	27.0 (26.0-28.0)	26.0 (26.0-28.0)	27.0 (26.0-29.0)	0.3094^‡^
Age (categorical)					
≤21	0	0	0	0	**
22-24	11 (4.8)	3 (3.6)	4 (5.6)	4 (5.4)	
25-27	133 (58.1)	52 (62.7)	43 (59.7)	38 (51.4)	
28-30	54 (23.6)	18 (21.7)	17 (23.6)	19 (25.7)	
31-33	18 (7.9)	7 (8.4)	6 (8.3)	5 (6.8)	
34-36	6 (2.6)	2 (2.4)	0	4 (5.4)	
≥37	7 (3.1)	1 (1.2)	2 (2.8)	4 (5.4)	
Sex					0.9429^*^
Male	108 (47.2)	39 (47.0)	33 (45.8)	36 (48.7)	
Female	121 (52.8)	44 (53.0)	39 (54.2)	38 (51.4)	
Degree					
Bachelor of Arts	46 (20.1)	19 (22.9)	11 (15.3)	16 (21.6)	0.4601^*^
Bachelor of Science	122 (53.3)	44 (53.0)	37 (51.4)	41 (55.4)	0.8869^*^
Master of Arts	2 (0.9)	0	1 (1.4)	1 (1.4)	0.5358^†^
Master of Science	20 (8.7)	6 (7.2)	4 (5.6)	10 (13.5)	0.1950^*^
MBA	2 (0.9)	1 (1.2)	1 (1.4)	0	0.7647^†^
MPH	2 (0.9)	1 (1.2)	0	1 (1.4)	1.0000^†^
PhD	1 (0.4)	0	1 (1.4)	0	0.3144^†^
MD	106 (46.3)	35 (42.2)	35 (48.6)	36 (48.7)	0.6413^*^
DO	94 (41.1)	37 (44.6)	29 (40.3)	28 (37.8)	0.6838^*^
DDS	7 (3.1)	1 (1.2)	4 (5.6)	2 (2.7)	0.2648^†^
DMD	13 (5.7)	6 (7.2)	2 (2.8)	5 (6.8)	0.4277^†^
Other	6 (2.6)	2 (2.4)	3 (4.2)	1 (1.4)	0.6020^†^
Marital Status					0.3420^†^
Single	130 (56.8)	46 (55.4)	45 (62.5)	39 (52.7)	
Married or with Significant Other	97 (42.4)	37 (44.6)	27 (37.5)	33 (44.6)	
Separated/Divorced	2 (0.9)	0	0	2 (2.7)	
Widowed	0	-	-	-	
Do you have children?					0.0126^*^
Yes	23 (10.0)	12 (14.5)	1 (1.4)	10 (13.5)	
No	206 (90.0)	71 (85.5)	71 (98.6)	64 (86.5)	
Primary Specialty					0.9990^*^
Family Med/General Prac	19 (8.3)	8 (9.6)	5 (6.9)	6 (8.1)	
Pediatrics	17 (7.4)	6 (7.2)	5 (6.9)	6 (8.1)	
Internal Med	53 (23.1)	18 (21.7)	19 (26.4)	16 (21.6)	
Obstetrics/Gynecology	18 (7.9)	6 (7.2)	6 (8.3)	6 (8.1)	
Surgery	22 (9.6)	7 (8.4)	7 (9.7)	8 (10.8)	
Emergency Medicine	42 (18.3)	17 (20.5)	13 (18.1)	12 (16.2)	
Dental	21 (9.2)	7 (8.4)	7 (9.7)	7 (9.5)	
Transitional	30 (13.1)	11 (13.3)	7 (9.7)	12 (16.2)	
Other	7 (3.1)	3 (3.6)	3 (4.2)	1 (1.4)	
JSE score median (IQR)	119.0 (109.0-128.0)	120.0 (111.0-130.0)	120.0 (108.0-128.0)	116.0 (105.0-126.0)	0.1553^‡^
Exhibit at least one manifestation of burnout (high EE or high DP)					0.0158^*^
Yes	70 (30.6)	20 (24.1)	18 (25.0)	32 (43.2)	
No	159 (69.4)	63 (75.9)	54 (75.0)	42 (56.8)	
MBI Emotional Exhaustion subscale score median (IQR)	16.0 (9.0-23.0)	15.0 (8.0-21.0)	15.5 (9.0-23.5)	16.5 (10.0-25.0)	0.4781^‡^
MBI Emotional Exhaustion subscale category					0.7885^*^
Low	145 (63.3)	56 (67.5)	46 (63.9)	43 (58.1)	
Moderate	42 (18.3)	13 (15.7)	14 (19.4)	15 (20.3)	
High	42 (18.3)	14 (16.9)	12 (16.7)	16 (21.6)	
MBI Depersonalization subscale score median (IQR)	5.0 (2.0-9.0)	4.0 (1.0-7.0)	5.0 (3.0-9.5)	7.0 (3.0-11.0)	0.0159^‡^
MBI Depersonalization subscale category					0.0935^*^
Low	121 (52.8)	52 (62.7)	38 (52.8)	31 (41.9)	
Moderate	51 (22.3)	17 (20.5)	16 (22.2)	18 (24.3)	
High	57 (24.9)	14 (16.9)	18 (25.0)	25 (33.8)	
MBI Personal Accomplishment subscale score median (IQR)	40.0 (36.0-45.0)	40.0 (37.0-44.0)	41.0 (36.0-46.0)	40.0 (33.0-44.0)	0.4853^‡^
MBI Personal Accomplishment subscale category					0.2245^*^
Low	127 (55.5)	46 (55.4)	41 (56.9)	40 (54.1)	
Moderate	61 (26.6)	26 (31.3)	20 (27.8)	15 (20.3)	
High	41 (17.9)	11 (13.3)	11 (15.3)	19 (25.7)	
Excluded from ESCI Analysis?					--
Yes	3 (1.3)	2 (2.4)	0	1 (1.4)	
No	226 (98.7)	81 (97.6)	72 (100)	73 (98.7)	
ESCI Achievement Orientation competency score (n=226) median (IQR)	4.5 (4.2-4.8)	4.5 (4.3-4.8)	4.7 (4.3-5.0)	4.5 (4.2-4.8)	0.1916^‡^
ESCI Adaptability competency score (n=226) median (IQR)	4.0 (3.8-4.5)	4.2 (3.8-4.5)	4.2 (3.8-4.6)	3.8 (3.5-4.3)	0.0123^‡^
ESCI Conflict Management competency score (n=226) mean ± SD	3.9 ± 0.6	4.0 ± 0.6	4.0 ± 0.6	3.8 ± 0.5	0.2066^¶^
ESCI Coach and Mentor competency score (n=226) median (IQR)	4.0 (3.7-4.3)	4.0 (3.7-4.3)	4.2 (3.7-4.5)	3.8 (3.3-4.3)	0.0407^‡^
ESCI Empathy competency score (n=226) median (IQR)	4.2 (3.8-4.5)	4.2 (4.0-4.5)	4.2 (3.8-4.8)	4.0 (3.7-4.3)	0.0081^‡^
ESCI Emotional Self-Awareness competency score (n=226) median (IQR)	4.0 (3.7-4.5)	4.2 (3.7-4.5)	4.2 (3.9-4.6)	3.8 (3.3-4.3)	0.0035^‡^
ESCI Emotional Self-Control competency score (n=226) median (IQR)	4.2 (3.8-4.7)	4.2 (4.0-4.7)	4.2 (3.8-4.7)	4.2 (3.7-4.5)	0.1453^‡^
ESCI Inspirational Leadership competency score (n=226) mean ± SD	3.7 ± 0.5	3.6 ± 0.5	3.7 ± 0.5	3.6 ± 0.6	0.2046^¶^
ESCI Influence competency score (n=226) median (IQR)	3.8 (3.5-4.3)	4.0 (3.5-4.2)	4.0 (3.8-4.5)	3.8 (3.3-4.2)	0.0015^‡^
ESCI Organizational Awareness competency score (n=226) median (IQR)	4.2 (3.8-4.5)	4.2 (3.8-4.5)	4.2 (4.0-4.7)	4.0 (3.7-4.3)	0.0346^‡^
ESCI Positive Outlook competency score (n=226) median (IQR)	4.0 (3.8-4.8)	4.3 (4.0-4.8)	4.1 (4.0-4.8)	4.0 (3.5-4.7)	0.0060^‡^
ESCI Teamwork competency score (n=226) median (IQR)	3.0 (2.8-3.2)	3.0 (2.8-3.2)	3.0 (2.9-3.2)	3.0 (2.8-3.2)	0.6238^‡^

Data presented are n(%) unless otherwise stated. Percentages might not add to 100% due to rounding. IQR=interquartile range; JSE=Jefferson Scale of Empathy; MBI=Maslach Burnout Inventory; EE=Emotional Exhaustion; DP=Depersonalization; ESCI=Emotional and Social Competency Inventory; SD=Standard Deviation *Chi-Square test was used to calculate p-value; †Fisher’s Exact test was used to calculate p-value; ‡Kruskal-Wallis test was used to calculate p-value; ¶ANOVA was used to calculate p-value; **Unable to compute p-value due to insufficient memory in computer

Furthermore, MBI-HSS Depersonalization (MBI DP) and MBI-HSS Personal Accomplishment (MBI PA) scores were also significantly different across specialties (χ^2^_(8, N=229)_ =15.93, p=0.0434, χ^2^_(8, N=229)=_20.80, p=0.0077, respectively). Bonferroni corrected pairwise comparisons did not reveal any significant differences between any of the specialties and EM. Pediatrics scored highest in MBI PA among specialties (Mdn=45); IM was lowest (Mdn=38) (Table 2).

Among the incoming residents assessed, there were no significant differences in any of the three MBI-HSS categories (Low/Moderate/High: Emotional Exhaustion, Depersonalization and Personal Accomplishment). It should be noted that the rate of presentation of at least one manifestation of burnout was high in every specialty: Tr (14, 46.7%), IM (19, 35.9%), EM (14, 33.3%), Other (2, 28.6%), OB/GYN (5, 27.8%), Dental (5, 23.8%), FM (4, 21.1%), Surgery (4, 18.2%), and Pediatrics (3, 17.7%). There were no differences in the 12 ESCI competencies between specialties.

Further analysis also identified differences in survey scores between genders. Of the respondents, 121 (52.8%) identified as female. When examining gender distribution across chosen specialties, there was a significant female predominance in both pediatrics (17, 100%) and OB/GYN (17, 94.4%) categories (χ^2^_(8, N=229)_ = 38.46, p<0.0001). First-year female residents demonstrated higher levels of empathy on the JSPE (Mdn=121) than their male counterparts (Mdn=115) (U=1080, p=0.0013) in addition to higher ESCI Empathy Competency Scores (Mdn=4.2 vs 4.0, U=10207, p<0.0001). Females also had higher (better) MBI PA scores (Mdn=41 vs 39, U=11214, p=0.0158), as well as higher scores in ESCI Achievement Orientation and Emotional Self-Awareness (Mdn=4.7 vs 4.3, U=10927, p=0.0123). Females scored lower in the ESCI Teamwork competency score (Mdn=3.0 (2.8-3.2) vs 3.0 (3.0-3.2), U=13519, p=0.0044). There was no significant difference in presence of burnout manifestations between the sexes, as measured by the MBI-HSS Emotional Exhaustion scale (female Mdn=14.5 vs male Mdn=16.0, U=11751, p=0.1816) and Depersonalization (female Mdn=5.0 (1.0-9.0) vs male Mdn=5.0 (3.0-10.0), U=13020, p=0.2295) subcategories (Table 3).

**Table 2:** Comparison of test scores between specialties

Survey Score	FM (n=19)	Pediatrics (n=17)	IM (n=53)	OB/GYN (n=18)	Surgery (n=22)	EM (n=42)	Dental 1.1.1 (n=21)	Transitional 1.1.2 (n=30)	Other (n=7)	c^2^	p-value
JSPE *median (IQR)*	127.0 (120.0-134.0)^†^	125.0 (117.0-131.0)	114.0 (105.0-121.0)	117.5 (114.0-127.0)	122.5 (109.0-132.0)	115.0 (109.0-127.0)	121.0 (113.0-128.0)	113.5 (103.0-122.0)	116.0 (98.0-125.0)	23.35	0.0029*
MBI EX *median (IQR)*	14.0 (6.0-19.0)	15.0 (7.0-20.0)	16.0 (10.0-25.0)	17.0 (15.0-26.0)	10.5 (9.0-22.0)	14.5 (10.0 (20.0)	13.0 (9.0-20.0)	21.0 (11.0-32.0)	13.0 (7.0-23.0)	12.09	0.1471*
MBI DP *median (IQR)*	2.0 (0.0-6.0)	3.0 (0.0-6.0)	6.0 (3.0-10.0)	4.5 (1.0-7.0)	4.0 (2.0-9.0)	7.0 (3.0-11.0)	4.0 (3.0-7.0)	7.0 (4.0-12.0)	3.0 (1.0-13.0)	15.93	0.0434*
MBI PA *median (IQR)*	43.0 (38.0-45.0)	45.0 (42.0-47.0)	38.0 (34.0-42.0)	40.0 (38.0-44.0)	39.0 (35.0-41.0)	41.0 (37.0-45.0)	42.0 (37.0-46.0)	38.5 (33.0-45.0)	40.0 (29.0-46.0)	20.80	0.0077*

*Kruskal-Wallis test used to calculate p-value for all comparisons †Bonferroni adjusted p-value was

**Table 3:** A gender-based comparison of respondent test scores

Characteristic	Male (n=108)	Female (n=121)	p-value
Age, years *median (IQR)*	27.0 (26.0-29.0)	27.0 (26.0-28.0)	0.1756
JSPE score *median (IQR)*	115.0 (105.0-124.0)	121.0 (111.0-130.0)	0.0013
MBI Personal Accomplishment subscale score *median (IQR)*	39.0 (34.5-43.0)	41.0 (36.0-45.0)	0.0158
MBI Emotional Exhaustion subscale score *median (IQR)*	14.5 (8.0-22.5)	16.0 (10.0-24.0)	0.1816
MBI Depersonalization subscale score *median (IQR)*	5.0 (3.0-10.0)	5.0 (1.0-9.0)	0.2295
ESCI Achievement Orientation competency score (n=226) *median (IQR)*	4.3 (4.0-4.8)	4.7 (4.3-4.8)	0.0123
ESCI Empathy competency score (n=226) *median (IQR)*	4.0 (3.7-4.3)	4.2 (4.0-4.7)	<.0001
ESCI Emotional Self-Awareness competency score (n=226) *median (IQR)*	4.0 (3.3-4.3)	4.2 (3.8-4.5)	0.0024
ESCI Teamwork competency score (n=226) *median (IQR)*	3.0 (3.0-3.2)	3.0 (2.8-3.2)	0.0044

Mann-Whitney U test was used to calculate all p-value

## Discussion

The concern for decreasing empathy and increasing burnout in medical residents is widely acknowledged.^6^^,^^8^^,^^9^^,^^16^ The current study focuses on whether there are correlations between resident demographics, specialty choice, baseline empathy, and burnout. A recent Chinese study found that empathy, distress, and psychological capital were not significantly correlated with age, gender, or specialization in a cohort of medical residents.^16^ Additionally, a multi-institutional study did not find significant differences across Surgery, Pathology, and Pediatric specialties in a global measure of EI.^17^ Conversely, another study found that training in Urology, Neurology, EM, Ophthalmology, and General Surgery were associated with higher relative risks of reported burnout symptoms, relative to IM.^18^ In the current study, EM interns demonstrated lower Empathy and higher Depersonalization scores, when compared to FM. There is limited experience in altering these scores. However, a study by Nelson and colleagues reported that patient experience simulation was an effective way to develop empathy in EM residents.^19^

In the current study, we found that incoming female residents tend to have more empathy and emotional intelligence than their male counterparts. Despite these findings, there was no significant difference in burnout levels between the sexes. Other demographic factors such as age, academic degrees obtained, marital status, and children were not linked to any significant change in scoring. Interestingly, our analyses found statistically significant differences between academic years in several emotional intelligence subcategories, rates of burnout indicators, and demographic information.

During the three years that this study was conducted (2015-2017), there were no major global, national, or local events, such as a natural disaster or the COVID-19 pandemic, that may correspond to the changes noted between entry years. As such, these data points warrant further investigation to determine if it is normal fluctuation due to the personality composition of each class, or if there is a systematic underlying cause that may be addressed to regulate the amount of burnout medical students experience upon graduation. Regardless of the underlying cause, the differences found between academic years indicate that each new class of interns merits individualized care plans to mitigate burnout and enhance empathy.

Transitional residents are somewhat unique in that they use their first year of residency training to gain a foundation in patient care before entering their eventual specialty. Given the percentage of Tr residents with a manifestation of burnout already present upon entry into the program (14, 46.7%), these residents may be at particular risk for increased burnout as their training continues. Identifying these individuals, engaging with them, and supporting their transition from one portion of their training to another may be key for GME to reduce burnout in incoming residents. Although not completely analogous to our Tr cohort, a Japanese study found that resident physicians who did not choose specialties at the beginning of the year were “significantly more susceptible to high depersonalization at the end of the year.”^20^

Although job burnout is most often discussed at the physician level, as the current study has indicated, burnout is prevalent at the graduate medical education level as well. With the aim of combating high levels of burnout experienced in traditional students and residents, a focus on interventions is needed. Ripp and colleagues studied two interventions for reducing burnout: practicing mindfulness-based stress reduction and participation in music, arts, advocacy work, and exercise.^21^ Both methods were shown to be effective.^21^ Another study created a mindfulness-based intervention for residents, which included two or three 1-hour mindfulness-based resilience training sessions performed during the resident didactic time.^22^ The mindfulness-based intervention did not have an effect on short-term stress and burnout levels. However, the follow-up surveys that were given to the residents found that there was a trend toward lower scores in stress and in burnout.^22^ Although this study was looking at short-term burnout levels, it does suggest that targeted initiatives during medical school may decrease stress and burnout levels over time.^22^ Stress reduction initiatives such as those referenced above may help mitigate the high levels of burnout present in medical school, seen here at the very beginning of residency.

This study is limited by including a convenience sample of incoming residents from a single health care network and represents a cross-sectional rather than a longitudinal assessment. With a large number of specialties and total sample size for this study, it is likely underpowered to detect some of the differences between certain specialties. Differences found among incoming residents within each specialty may be attributable to differing undergraduate medical education experiences, but this has yet to be demonstrated. A cohort of second-year residents surveyed during their fourth year of medical school showed that higher anxiety and lower empathy in medical school were associated with increased risk for reported burnout during residency two years later.^18^ The effect of educational interventions and individual support during a residency on levels of empathy, EI, and burnout warrant further investigation. Additional limitations of this study include self-reporting and social desirability biases. These biases could diminish the extent to which new residents may report what they perceive as negative personality traits.

## Conclusions

Although measures of burnout are low in most incoming residents regardless of specialty, within each specialty there are entering residents with high levels of burnout even prior to starting residency. Males and females did not differ in levels of burnout, yet females across specialties demonstrated higher levels of empathy and personal accomplishment. Monitoring burnout levels and providing individualized support to new residents to help address burnout at an early stage in their GME may help to improve overall graduation and performance rates among these new medical professionals. Future studies should examine residents longitudinally to determine if burnout, EI, and empathy change over the course of a typical residency. In addition, educational techniques could be compared to determine if there are methods that help to mitigate these effects. Future investigations might also include surveys from residents in multiple institutions and should evaluate programs designed to ameliorate symptoms of burnout.

### Acknowledgments

Authors would like to acknowledge the research operations management of Alex Rosenau, DO, Sofia M. Murillo, BS, PA-S, and Andrew J. Ferdock, BS, for their support. The authors would also like to acknowledge Shae Duka, MPH, for her assistance with data analysis.

### Conflict of Interest

The authors declare that they have no conflict of interest.
